# Role of the UNC13 family in human diseases: A literature review

**DOI:** 10.3934/Neuroscience.2023029

**Published:** 2023-12-06

**Authors:** Ubaid Ansari, Vincent Chen, Romteen Sedighi, Burhaan Syed, Zohaer Muttalib, Khadija Ansari, Fatima Ansari, Denise Nadora, Daniel Razick, Forshing Lui

**Affiliations:** California Northstate University College of Medicine, USA

**Keywords:** unc13, neurotransmission, amyotrophic lateral sclerosis, frontotemporal dementia, familial hemophagocytic lymphohistiocytosis, autism spectrum disorders, oral squamous cell carcinoma, hepatocellular carcinoma, Alzheimer's disease

## Abstract

This literature review explores the pivotal roles of the Uncoordinated-13 (UNC13) protein family, encompassing UNC13A, UNC13B, UNC13C, and UNC13D, in the pathogenesis of various human diseases. These proteins, which are evolutionarily conserved and crucial for synaptic vesicle priming and exocytosis, have been implicated in a range of disorders, spanning from neurodegenerative diseases like amyotrophic lateral sclerosis (ALS) and frontotemporal dementia (FTD) to immune-related conditions such as familial hemophagocytic lymphohistiocytosis (FHL). The involvement of UNC13A in neurotransmitter release and synaptic plasticity is linked to ALS and FTD, with genetic variations affecting disease progression. UNC13B, which is closely related to UNC13A, plays a role in autism spectrum disorders (ASD), epilepsy, and schizophrenia. UNC13C is implicated in oral squamous cell carcinoma (OSCC) and hepatocellular carcinoma (HCC), and has a neuroprotective role in Alzheimer's disease (AD). UNC13D has an essential role in immune cell function, making it a key player in FHL. This review highlights the distinct molecular functions of each UNC13 family member and their implications in disease contexts, shedding light on potential therapeutic strategies and avenues for future research. Understanding these proteins' roles offers new insights into the management and treatment of neurological and immunological disorders.

## 1. Introduction

In recent years, the study of molecular mechanisms underlying various human diseases has garnered significant attention within the scientific community. In particular, neurological and immunological disorders have been the focus of extensive research efforts, aiming to uncover the intricate pathways and molecules that govern disease initiation, progression, and manifestation. Among the key players identified in these contexts, members of the uncoordinated-13 (UNC13) protein family have emerged as pivotal contributors, influencing a range of physiological processes across diverse cell types [1]. Collectively known as the UNC13 family, UNC13A, UNC13B, UNC13C, and UNC13D exhibit distinct functional roles within various cellular contexts and have been implicated in the pathogenesis of multiple diseases. The UNC13 family proteins are evolutionarily conserved members of the priming factor family that play essential roles in synaptic vesicle priming and exocytosis, as well as other cellular processes such as immune responses [2]. The genetic variations and dysregulation of these proteins have been associated with an array of disorders, thus highlighting their indispensable contributions to human health [2]. This review aims to comprehensively examine the role of each UNC13 family member—UNC13A, UNC13B, UNC13C, and UNC13D—in different diseases, thereby shedding light on their distinct molecular functions and their implications in pathological contexts.

UNC13A, which is predominantly expressed in the nervous system, has been implicated in many neurodegenerative disorders and neurological conditions such as amyotrophic lateral sclerosis (ALS) and frontotemporal dementia (FTD) due to its involvement in neurotransmitter release and synaptic plasticity [3]. UNC13B, which is closely related to UNC13A, has shown its significance in autism spectrum disorders (ASD), partial epilepsy, and schizophrenia [4]. Additionally, it has been associated with neurodegenerative disorders and immune-related diseases. Although less studied compared to its counterparts, UNC13C has been implicated in several disease states such as oral squamous cell carcinoma (OSCC) [5] and hepatocellular carcinoma (HCC) [6]. Moreover, UNC13C is believed to be a protective gene against disease progression in Alzheimer's dementia (AD) [7]. On the other hand, UNC13D is prominently recognized for its contribution and importance in immune cell function, particularly in T-cell mediated cytotoxicity. Mutations in UNC13D have been linked to familial hemophagocytic lymphohistiocytosis (FHL), thereby highlighting its role in regulating immune responses [8]; moreover, associated mutations can lead to defective cytotoxic granule exocytosis in natural killer (NK) cells and cytotoxic T lymphocytes (CTLs), contributing to the dysregulated immune responses seen in FHL [9]. As the understanding of UNC13 family proteins' roles in disease pathogenesis advances, potential therapeutic avenues may arise. Targeting these proteins could provide novel strategies for managing and treating a spectrum of neurological and immunological disorders. This review aims to consolidate the current knowledge surrounding UNC13A, UNC13B, UNC13C, and UNC13D, highlighting their unique contributions to various diseases and illuminating potential avenues for future research and therapeutic intervention.

**Table 1.** Function and associated diseases of UNC13A, B, C, D.

**UNC13**	**A**	**B**	**C**	**D**
Function	Primes synaptic vesicles and aids in neurotransmitter release at nerve terminals [12]	Synaptic vesicle priming and fusing [21]	Mediation of excitatory neurotransmitter release via priming and stabilizing vesicular complexes [28]	Immune responses [40]
Associated Diseases	Neurodegenerative diseases: ALS, FTD [3]	ASD, Partial Epilepsy, Schizophrenia [1]	OSCC, HCC; protective against AD [5],[6],[7]	FHL [8]

## 2. Review

### 2.1. Pathophysiological mechanisms in UNC13A

The UNC13A gene has recently gained significance as a risk locus in the pathogenesis of ALS and FTD [3]. Specifically, a single nuclear polymorphism within the UNC13A, rs12608932, has been identified to potentiate the acquisition and progression of both ALS and FTD [10]. UNC13A exerts its influence through its reciprocal relationship with transactive response DNA-binding protein 43 (TDP-43), which is a DNA-binding protein that inhibits the inclusion of cryptic exons in UNC13A gene [11]. Polymorphisms within UNC13A, such as rs12608932, have been found to deplete TDP-43, ultimately leading to decreased expression of UNC13A and neurotransmitter release [11]. As such, UNC13A polymorphisms have been identified as a target of interest in the treatment of ALS and FTD.

The translated UNC13A protein plays an essential role in vesicle maturation and neurotransmitter release at the presynaptic nerve terminals. After localizing to the active zone of the presynaptic density, UNC13A primes and facilitates the fusion of the vesicle with the neuronal membrane [12]. Experiments performed on the mouse variant Munc13 have provided illuminating details into the mechanistic function of the protein. As expected, mutations in Munc13 disrupt neurotransmitter release, but notably, deficiencies in Munc13 proteins do not impair electric conduction, calcium influx from voltage-gated calcium channels, synaptic integrity, or vesicle glutamate concentrations [13]. Rather, research suggests that Munc13 interacts with soluble NSF attachment protein receptor (SNARE) proteins (namely syntaxin-1) and promotes the assembly of a stable complex, thus resulting in active vesicles capable of fusing with the synaptic membrane [12]. More recently, studies have revealed the link between ALS and FTD and single nucleotide polymorphisms (SNP) within UNC13A [14]. The variant integrity of UNC13A is directly dependent on the functionality of TDP-43, which is a nuclear protein that plays an instrumental role in splicing RNA and repressing the inclusion of cryptic exons [15],[16]. In this way, TDP-43 prevents the degradation of the mRNA by inhibiting the introduction of frameshifts and stop codons in the resulting mRNA [11],[14],[16].

Crucially, it has been found that UNC13A SNPs in some patients led to the depletion of TDP-43, potentiating loss of UNC13A functionality [11]. One such SNP, rs12608932, has an especially strong association with FTD and ALS [10]. A study performed in 2023 demonstrated that proteinopathy in TDP-43, as expected, is a signature characteristic in more than 97% of ALS cases and 45% of FTD cases [14]. Interestingly, rs12608932 is a strong determinant of shorter survival times for ALS, but not FTD [17]. Genome-wide association studies have demonstrated that homozygosity for the C-allele at rs12608932 leads to shorter survival times in ALS patients when compared with either homozygous (A/A) or heterozygous (A/C) individuals [3]. One study also demonstrated that there was up to 33.0 months difference in life expectancy for carriers of the rs12608932 genotype [17]. Carriers of at least one risk allele had a 11.7 month shorter median survival time as compared to wild-type individuals, even after adjusting for age [17]. However, there was no association between the presence of rs12608932 and survival in FTD.

Since ALS presents with a large genetic heterogeneity, it is expected that treatments may benefit different genetic subgroups differently. As identified in an exploratory meta-analysis, patients with homozygous C-alleles at SNP rs12608932 showed a statistically noteworthy benefit being treated with lithium carbonate. Another published report documents 44 patients studied, divided into 28 taking only riluzole and 16 taking riluzole paired with lithium carbonate [18]. Riluzole is a benzothiazole that works to change the activity of natural bodily substances that have an influence on nerves and muscles, thus extending the survival time of patients but not curing them [19]. Fifteen months after entry, the 16 subjects given both riluzole and lithium carbonate had a 100% survival rate, whereas the group of riluzole-only patients had 71% survivability. Faults can be found within this trial on several levels; however, this and other studies provide enough ground for lithium carbonate to be explored as a potential treatment.

While this provides a certain level of motivation to further use lithium carbonate as a potential treatment, there are contrary studies to suggest that lithium carbonate need not be an area of focus for ALS research. A large analysis of three separate studies involving lithium carbonate's effect on 518 patients provided evidence that only a certain subgroup can gain a benefit from the administration of lithium carbonate [20]. This suggests that lithium carbonate can only be tested and further supplied as a treatment to those carrying the UNC13A C/C genotype. Additionally, this study generally illuminates the importance of factoring genes into the equation when discussing treatments for ALS [20]. As for the study itself, subjects were divided by 261 patients receiving lithium carbonate and 257 serving as a control, then subdivided to 174 on placebo and 83 on a subtherapeutic dosage of lithium carbonate. ALS patients carrying the UNC13A genotype account for less than 20% subjects of this study; this may indicate an imbalance in results regarding the effectiveness among groups who are actually eligible [20]. Regardless, when solely considering the responsive subjects who were identified to be carrying UNC13A, the survivability rate of patients was shown to have improved from 40% to 69.7% [20]. The incorporation of genetic data in these trials displays the importance of identifying UNC13A in ALS patients, so that more targeted trials may take place in the future, thereby providing lithium carbonate specifically to carriers and further subdividing the genotype. At the same time, this would allow for the tailoring of other potential treatments and tests for noncarriers of other genotypes, as ALS has been proven to affect different genotypes with variation [20]. This makes it very possible that ALS needs to be treated with individually considered therapies depending on the genetic makeup of patients.

### 2.2. Pathophysiological mechanisms in UNC13B

UNC13B gene is a homolog of the UNC13 gene sequence and encodes the presynaptic protein mammalian uncoordinated 13-2 (Munc13-2), which is highly expressed throughout the brain but markedly in the cerebral cortex and is thought to play a role in neuronal excitability. The gene product plays an essential role in synaptic vesicle priming through the assembly and arrangement of synaptic vesicles at the nerve terminals, which are readily releasable upon arrival of an action potential [4]. The UNC13B gene is a homolog of the UNC13A gene. Other possible homologs include UNC13C and UNC13D. UNC13B is thought to enable GTP dependent protein binding activity, calmodulin activity, and syntaxin-1 binding activity [21]. It acts upstream of several processes including chemical synapse transmission, innervation, and regulation of neuronal plasticity. This homolog, while being expressed largely in the central nervous system, is also found in the eye, respiratory system, genitourinary system, and the alimentary canal [4].

The gene product of UNC13B is a target of the diacylglycerol second messenger pathway system and helps to regulate synaptic vesicle release at nerve terminals [22]. Along with UNC13A, the gene product of 13B helps to facilitate neuronal dense core vesicle fusion and controls the efficiency of vesicle release [22]. Additionally, UNC13B is important in hippocampal synaptic plasticity and transmission [4]. Munc13-2 is needed for normal release probability at the hippocampal mossy fiber synapses. Paired pulse and frequency were strongly increased; however, mossy fiber long-term potentiation was unaffected in the absence of Munc13-2.

Additionally, the UNC13B gene has been linked to the disease formation of partial epilepsy. Patients with the variant types of UNC13B gene products had partial epilepsy and/or febrile seizures [4]. Moreover, a few patients studied were found to have brain abnormalities, especially in the hippocampal region. Regardless, all patients had favorable outcomes without difficulties in learning or other developmental issues. The identified variants in epilepsy patients had mutations in the UNC13B gene, which included one nonsense variant, two variants at or around a splice site, one heterozygous missense variant, and four missense variants which were seen in the familial cases of epilepsy [4]. Additionally, a knockdown of UNC13B in Drosophila resulted in an increased rate of seizures and their associated durations compared to wild-type flies [23]. Electrophysiological studies performed in the study showed that excitatory neurons in flies that were UNC13B deficient exhibited an increased excitability. The results seen in the Drosophila study and the seizure study indicated that UNC13B is potentially associated with partial epilepsy [23]. The frequent daily seizures and hippocampal abnormalities were seen in patients studied, but ultimately using medications and other treatments, UNC13B was found to have no debilitating effects on affected patients. Additionally, UNC13B variants were seen to have blurring of neurons in the mushroom body, partial destruction of the central complex, and gamma lobe mutations in the brains of the epileptic patients [23]. Overall, UNC13B seems to have a link in neuronal excitability and the development of seizures in epileptic patients.

UNC13B substitutions are also linked to the increased risk of nephropathy in patients with Type 1 Diabetes (T1D). Specifically, substitution of G/T 1 in intron 1 was seen to increase the risk of nephropathy in patient populations [24]. The mutations were identified through the sequencing of various regions of DNA including a promoter, an exon, and the flanking intron gene regions. Only one SNP, namely rs2281999, which is located in the UNC13B gene, was seen to be significantly associated with nephropathy after correction for multiple testing was performed [21]. Conducting an analysis of 21 other markers, which identified the variability of the UNC13B gene, showed consistent associations of the SNP rs13293564 with nephropathy in the three populations [21]. In the presence of hyperglycemia, which is an event occurring early in the development of nephropathy, UNC13B mediates apoptosis in glomerular cells. Therefore, UNC13B polymorphisms are implicated in nephropathic damage in type T1D patients through the loss of glomerular cells and reduced renal function in affected patients [25]. The possible link of UNC13B to multiple disease processes outside the brain shows how essential gene products of this gene are in the body.

UNC13B polymorphisms were also identified in autism and some schizophrenic patients. Studies of multiplex families revealed a role in the genetic presentation of the disease [26]. Whole-exome sequencing (WES) in two affected and one unaffected individual of a multiplex family with 10 affected individuals was performed alongside a follow-up resequencing of the unc-13 human homolog B; it was found that certain polymorphisms were seen to be greater in schizophrenia families [27]. The five rare sequences seen were SMARCA5, PDE1B, TNIK, SMARCA2, and FLRT, which were shared among all affected members and predicted to be damaging to the patients [27]. Variants from the SMARCA5 and PDE1B groups were inherited from the unaffected father, whereas variants from TNIK, SMARCA2, and FLRT were inherited from the unaffected mother, and disease incidence was also seen in the third generation [24]. Moreover, all five variants were transmitted by an affected mother to her affected son. With support at the variant gene levels, the multiple heterozygous protein sequences altering variants identified in disease related genes may help contribute to the development of disease in families with a history of schizophrenia. Therefore, UNC13B seems to play important roles within the properly functioning body, and polymorphisms and mutations may lead to the development and incidence of several different acute diseases.

### 2.3. Pathophysiological mechanisms in UNC13C

The role of Unc-13, which is a related gene, in facilitating neurotransmission was first discovered in C. Elegans, with deficient worms exhibiting altered movements. Further research was conducted using the mammalian variant in mice (Munc13), which is evolutionarily conserved and orthologous to the human variant UNC13A [3]. Munc13 deficiency in mice was found to induce total paralysis and fatality before birth, with the neuromuscular synapses of the subjects expressing fewer vesicles capable of exocytic fusion and a marked reduction in neurotransmitter release, thus resulting in impaired synapse formation [12]. Deficient mice did not exhibit significant changes to electric conduction, abnormal calcium influx from voltage-gated Ca^2+^ channels, synaptic damage, vesicular glutamate concentrations, or aberrations in postsynaptic glutamate receptors. Despite this, Munc13 deficiency was shown to result in a corresponding loss in excitatory neurotransmitter release, thereby leaving a large portion of synapses functionally silent. Successfully forced stimulation of these incompetent synapses in Unc-13 deficient subjects was possible with α-latrotoxin, which is a presynaptic neurotoxin, indicating that the gene does not directly participate in vesicle fusion [13]. Further research suggests that Unc-13 interacts with the vesicle SNARE proteins (namely syntaxin-1) and promotes the assembly of a stable complex, thereby resulting in active vesicles capable of fusing with the synaptic membrane [12].

A member of the UNC13 protein family, unc-13 human homolog C (UNC13C), has been linked to mediation of excitatory neurotransmitter release through the priming and stabilizing of vesicular complexes, thereby allowing glutamatergic vesicles to dock at the plasma membrane prior to exocytosis [28]. Extensive insight on the precise structure and function of UNC13C has been gleaned from the study of the orthologous mammalian gene Munc13-3. In rodent brains, Munc13-3 is largely expressed in the presynaptic terminals of the granular cells (GCs) and Purkinje cells of the cerebellum [29]. Unlike Munc13-1 deficient mice, Munc13-3 double knockout mice were observed to possess an intact cerebellar structure, including synaptic vesicle density and distribution and an overall brain morphology similar to the wild-type controls; nevertheless, these mice had compromised synaptic transmission at GC-Purkinje junctions, with a decreased glutamate release probability (*p_r_*) in their cerebellar tissue [29]. Further research on the association between Munc13-3 and synaptic vesicle release demonstrated that *p_r_* is nearly cut in half at cerebellar granule-basket cell (GC-BC) synapses when Munc13-3 is deleted, suggesting the gene's potential role in “super priming” either via increasing sensitivity of release components to Ca^2+^ or through repositioning of the vesicles for enhanced coupling to the Ca^2+^ influx [30]. It has been suggested that super priming by Munc13-3 may involve regulation and localization of Ca_v_2.1 and Ca_v_2.2 channels to facilitate nanodomain-coupled vesicle release at the presynaptic terminal of cerebellar parallel fibers, with deficient synapses failing to develop this more sensitive, targeted coupling apparatus [31]. This finding was associated with impaired motor learning in higher trials for Munc13-3 deficient mice when wild-type and knockout groups were tested with progressively challenging motor tasks [29]. Munc13-3 deficiency was not associated with any abnormal changes to visual/olfactory function, movement activity, and spatial/working memory, potentially due to the sufficiency of Munc13-1 and Munc13-2 for these functions; however, mutant mice did show significantly reduced acoustic startle responses [32].

In humans, UNC13C deficiency has been implicated in several disease states, including OSCC, with single nucleotide variants identified in as many as 12% of OSCC patients according to one study, which is a significantly higher rate than in the general population. It was unclear what role UNC13C played in tumor pathogenesis or whether a deficiency could be associated with the predisposition to nicotine addiction and tobacco use in OSCC [33]. However, further research has indicated that tumor cell lines in OSCC patients express UNC13C at significantly lower levels than surrounding healthy tissue, with low expression levels also being correlated with poor prognosis for survival in this patient population. Significantly decreased UNC13C mRNA and protein expression in OSCC cell lines was also reproduced in a study on the protein's anti-cancer effects [5]. UNC13C overexpression was shown to repress key proteins required for the epithelial-mesenchymal transition, which is a critical process in early tumor cell migration and metastasis, suggesting that UNC13C may directly act as a tumor suppressor [34]. Moreover, UNC13C silencing has been shown to repress the activity of the miR-96-5p inhibitor, resulting in lowered apoptotic tendency and susceptibility to radiotherapy in OSCC cells, further establishing the role of UNC13C in tumor suppression [5]. In contrast, recent research on the role of UNC13C in the prognosis of HCC has suggested that high cytoplasmic expression of the protein may be paradoxically associated with increased American Joint Committee on Cancer (AJCC) T-staging, alcohol consumption, and a lower overall survival prognosis in HCC patients, thereby indicating possible oncogenic function acquired by UNC13C [6].

Genetic studies have also correlated a lack of UNC13C expression with neurological and neuromuscular deficits. UNC13A and UNC13C for example, were among a group of exocytosis-related genes significantly downregulated in patient populations with ALS; downregulation of other vesicle-fusion regulatory proteins in ALS, such as SNAP25, is linked to the elevated intraneuronal calcium concentration and the resulting glutamate excitotoxicity associated with the pathophysiology of ALS [35]. Even in non-disease states, significant differential expression of UNC13C was observed when comparing healthy and frail elderly subjects, underscoring the link between UNC13C and neuromuscular wasting [36]. In AD, UNC13C is believed to be a protective gene against disease progression as its expression is decreased in the vulnerable CA1 hippocampal region but conserved in the less affected CA3 region, thereby suggesting that the loss of UNC13C and other protective gene products may accelerate the greater neuronal dysfunction observed in CA1 compared to CA3 in AD patients [7]. One study investigating inherited gene mutations in AD found in-frame UNC13C deletions for two dementia patients, whereas this mutated variant was missing for two healthy family members; however, the small sample size and presence of confounding variables such as the presence of apolipoprotein E (APOE) ɛ4 in these subjects warrants more research on the nocuous effects of the UNC13C mutation in AD [37]. Although certain anticancer medications such as Lenvatinib have been shown to repress the relative expression of UNC13C in treatment groups [38], the use of genetic therapy and targeted medication to reverse UNC13C deficiency has been largely unexplored thus far and remains a potential avenue for the treatment of specific cancers and neurological disorders.

### 2.4. Pathophysiological mechanisms in UNC13D

As previously mentioned, the UNC13 family of proteins plays vital roles in synaptic vesicle priming and exocytosis. The UNC13D gene specifically encodes for the Munc13-4 protein. Unlike the other proteins in the Munc13 families, which arise from the other UNC13 homologues, Munc13-4 lacks an N-terminal phorbol ester-binding C1 domain. This is an important regulatory domain for neuronal priming. However, Munc13-4 does share two C-terminal C2 domains that are involved in the process of exocytosis and vesicle priming, which is common across all the Munc13 homologues [39]. The protein is essential for CTLs and NK cells of the immune system. It facilitates the fusing of the cytotoxic granules with the plasma membrane of either the CTL or the NK cell, thereby enabling the proper release of proteins such as perforin and granzymes to promote cell death [40]. Although the exact process is still being researched, the protein is involved in the intracellular transport and exocytosis of these lytic granules containing these glycoproteins that attach to the targeted cell as part of the immune response. Exactly how Munc13-4 is activated to bind the membrane with the granule is unknown [41].

With these functions in mind, it is apparent that UNC13D and the protein it encodes for is vital in the immune response. With the help of major histocompatibility complex (MHC) class 1 molecules in identifying infected cells, CTLs and NK cells can induce cell death, thereby protecting the body against spreading of infection to healthy cells [9]. Munc13-4 plays an important role helping the CTLs and NK cells do their job effectively, and mutations in the UNC13D gene can show the extent of this. Mutations or dysfunctions in the gene can lead to a rare autosomal recessive disease called familial hemophagocytic lymphohistiocytosis type 3 (FHL3). FHL is characterized by overactivation and excessive proliferation of T cells and macrophages, which can lead to damage of organs such as spleen, liver, and brain after infiltration from the immune cells [8]. The characteristic hyperinflammation leads to hypercytokinemia and lymphohistiocytic proliferation. This widespread inflammation leads to the failure of multiple organs, and mortality rates are between 42% and 88%. Common symptoms include fever, splenomegaly, cytopenia, liver dysfunction, and even neurologic abnormalities. Although the disease primarily affects infants, it can be found in all age groups. Essentially the disease is identified by the dysregulation of lymphocyte activity, as well as causative mutations in genes such as PRF1, STX11, STXBP2, or UNC13D [42].

The FHL3 subtype is characterized by its mutations in the UNC13D gene. The gene consists of 32 coding exons, which encode 1,090 amino acids for the Munc13-4 protein [43]. In a 2010 study performed in South Korea, 40 patients that were suspected to have FHL were recruited. Of the 40, nine patients were identified with FHL mutations. Of those nine, eight had a mutation in UNC13D. The mutations were all of a deleterious nature, with the presence of recurrent splicing mutations. Despite the small sample size of FHL patients, the data showed that UNC13D is a major FHL gene among Koreans [44]. It has been found that at least 112 different mutations of UNC13D have been reported as a cause of FHL3. This includes variations outside of the exons and splice sites of the gene. In one study, it was found that an intronic mutation of the UNC13D gene was found to impair its transcription, perhaps by interfering with either a transcription factor binding site or enhancer element [45]. The various mutations of the gene led to deactivated Munc13-4, and in FHL3 patients with these mutations, a decrease of lytic granules fusing with the CTL plasma membrane was noted [46]. It is clear that impaired transcription, and thus impaired function of Munc13-4, can lead to many problems impacting the immune system, including disorders such as FHL3. Therapies for this disorder include chemoimmunotherapy to help with the active symptoms, as well as allogeneic hematopoietic stem cell transplantation [47]. With further study, experiments, and improvements in gene therapy technology, the causative mutations in the UNC13D gene can be targeted, and new avenues of treatment can be uncovered for FHL.

## 3. Conclusion

The UNC13 family of proteins encompass a critical set of molecules involved in diverse roles in cells, and each member of this family (UNC13A, UNC13B, UNC13C, and UNC13D) hold distinct roles in terms of different diseases. Through exploring molecular functions and certain pathological contexts, information on each protein has been obtained for this literature review. To start, UNC13A is involved in neurodevelopmental and neurological disorders which highlights its significance in neurotransmitter release and synaptic plasticity, thus offering potential therapeutic avenues for conditions such as ALS and FTD [3]. UNC13B impacts ASD, partial epilepsy, and schizophrenia. Moreover, it takes an active role in immune-related diseases and neurodegenerative disorders [4]. The UNC13B gene encodes the presynaptic protein Munc13-2, which plays a crucial role in synaptic vesicle priming and fusion. This gene is highly expressed in the brain, especially in the cerebral cortex, where it influences neuronal excitability [4]. UNC13C plays a role in tumor suppression in the pathogenesis of OSCC [5] and an oncogenic role in the pathogenesis of HCC [6]. Additionally, it has been identified as a protective gene in AD [7]. Furthermore, neurological and neuromuscular deficits have been linked to a decreased expression of this gene. The final protein, UNC13D, has a critical role in cytotoxic granule exocytosis and has been linked to severe immune disorders like FHL [8].

The knowledge gained through our research on the proteins within the UNC13 family and their roles in disease pathogenesis allows for new avenues into possible management strategies and treatments. There is a greater potential for drug development as the study of proteins, their interactions with molecules, and effects on certain cellular processes continues. Looking into these specific proteins will lead to stronger approaches in both managing and treating a wide spectrum of immunological or neurological disorders or both in tandem. In addition, the research conducted from concentrating on the UNC13 family proteins could add to the more extensive comprehension of cellular processes past what is currently known on direct functions, which, in turn, will allow for more research in diverse fields of medicine. This literature review on the UNC13 family investigated the various roles, functions, and necessary insights that will pave the way for ongoing research of these proteins, which will inevitably improve healthcare.
